# ﻿*Aeschynanthussmaragdinus* F.Wen & J.Q.Qin (Gesneriaceae), a new species from Yunnan Province, China

**DOI:** 10.3897/phytokeys.220.98040

**Published:** 2023-02-24

**Authors:** Jia-Qi Qin, Rui-Feng Li, Yan-Ping Pang, Fang Wen

**Affiliations:** 1 Shanghai Botanical Garden, CN-200231, Shanghai, China Shanghai Botanical Garden Shanghai China; 2 Gesneriad Conservation Center of China (Shanghai), CN-200231, Shanghai, China Gesneriad Conservation Center of China Shanghai China; 3 College of Tourism and Landscape Architecture, Guilin University of Technology, CN-541006, Guilin, China Guangxi Institute of Botany, Guangxi Zhuang Autonomous Region and Chinese Academy of Sciences Guilin China; 4 Guangxi Key Laboratory of Plant Conservation and Restoration Ecology in Karst Terrain, Guangxi Institute of Botany, Guangxi Zhuang Autonomous Region and Chinese Academy of Sciences, CN-541006, Guilin, China Guilin University of Technology Guilin China; 5 National Gesneriaceae Germplasm Resources Bank of GXIB, Gesneriad Committee of China Wild Plant Conservation Association, Gesneriad Conservation Center of China (GCCC), Guangxi Institute of Botany, Guilin Botanical Garden, Guangxi Zhuang Autonomous Region and Chinese Academy of Sciences, CN-541006, Guilin, China Guangxi Institute of Botany, Guilin Botanical Garden, Guangxi Zhuang Autonomous Region and Chinese Academy of Sciences Guilin China

**Keywords:** *Aeschynanthuschiritoides*, Didymocarpoideae, Flora of Yunnan, taxonomy

## Abstract

*Aeschynanthussmaragdinus* F.Wen & J.Q.Qin, a new species of Gesneriaceae from the monsoon rain forest in Mangbang township, Tengchong City, Yunnan Province, China, is described and illustrated here. It morphologically resembles *A.chiritoides* C.B.Clarke in size, shape and hairs on the leaf blades. But it can easily be distinguished from the latter by the green corolla limb with brownish-red to maroon lower lobes. At the same time, the hairs of the pedicel and calyx lobes, the length of the staminode and the size of the seed grain can also help distinguish both. It is provisionally assessed as Data Deficient (DD), according to the IUCN Red List Categories and Criteria, because field surveys for this new taxon have not been completed.

## ﻿Introduction

*Aeschynanthus*Jack (1823) belongs to Subtribe Didymocarpinae G.Don, Tribe Trichosporeae Nees, subfamily Didymocarpoideae Arn., of the family Gesneriaceae Rich. & Juss. (Weber et al. 2013). In horticulture, this group is commonly called lipstick vine. All species of this genus are epiphytic and lithophytic plants. Approximately 160 known species are distributed in India, New Guinea, Solomon Islands, Southeast Asia, southern & southwestern China, and Sri Lanka (Weber 2004; Middleton 2007, 2009, 2016; Wei 2018; Olimpos and Mansibang 2021; Wei et al. 2022). Like many genera of Gesneriaceae, the genus is widespread, but local endemism at the species level is high (Mendum et al. 2001). There are currently 35 known species of *Aeschynanthus* in China (Li and Wang 2005; Hu et al. 2020). The Flora Pan-Himalaya project has developed a preliminary catalog of 32 species (Chen 2019). Many species are endemic to China (Wang et al. 1998). The corolla color of the *Aeschynanthus* species is variable, so it is challenging to describe. However, the characteristic of corolla color gradations is still important for the taxonomy of *Aeschynanthus*. The fruit type of this genus is a long narrow capsule (Wang et al. 1998; Middleton 2007, 2009).

During a field trip to Tengchong, Yunnan for Gesneriaceae investigation in 2017, an unknown pendulous *Aeschynanthus* species was found on tree trunks in a monsoon rain forest in Mangbang township, Tengchong city, Yunnan, China. This plant did not match any known species of *Aeschynanthus* in China or the neighboring countries. Some living plants were introduced and cultivated at the Gesneriad Conservation Center of China (GCCC), the National Gesneriaceae Germplasm Bank of GXIB, and the Shanghai Botanical Garden for further research. A comparison of living plants with type specimens and protologues of all known species of *Aeschynanthus* from China and neighboring countries led to the determination that these specimens neither fit the existing protologues nor conform to the known type specimens. The tiny shape and texture of the leaves make them very particular and similar to *A.chiritoides* C.B.Clarke (Middleton 2009; Cen et al. 2017). However, a combination of characteristics quickly distinguished it from the latter, especially in some important characters, viz. phyllotaxis variation, pedicel and calyx lobes indumentum, corolla and limb lobes color, staminode length, disk, seed grain size, and seed appendages length. We confirmed that it represents a new species of *Aeschynanthus* and describe it here.

## ﻿Taxonomic treatment

### ﻿ Aeschynanthus smaragdinus

Taxon classificationPlantaeLamialesGesneriaceae

F.Wen & J.Q.Qin sp. nov.

B42DE371-BBB0-5E3A-90B2-FA654E8B7B70

urn:lsid:ipni.org:names:77314715-1

Figs 1 , 2 , 3 , 4

#### Diagnosis.

The new species resembles *Aeschynanthuschiritoides* C.B.Clarke (Fig. 5) in leaf blades size, shape and indumentum, but can be easily distinguished from the latter by its pedicel densely erect glandular-pubescent (*vs.* densely villous), calyx lobes adaxially nearly glabrous and abaxially erectly glandular puberulent (*vs.* adaxially and abaxially glandular- or eglandular-villous or a mixture of both), corolla pale yellowish green to greenish (*vs.* white or slightly yellowish or greenish with a few thin purple lines), corolla limb upper lobes green and lower lobes brownish red to maroon (*vs.* all lobes white or slightly pale green with pale purplish lines), seed grain ca. 1.0× 0.5 mm (*vs.* 1.2–3 × ca. 0.3 mm). Detailed morphological comparisons with *A.chiritoides* are provided in Table 1.

**Figure 1.**
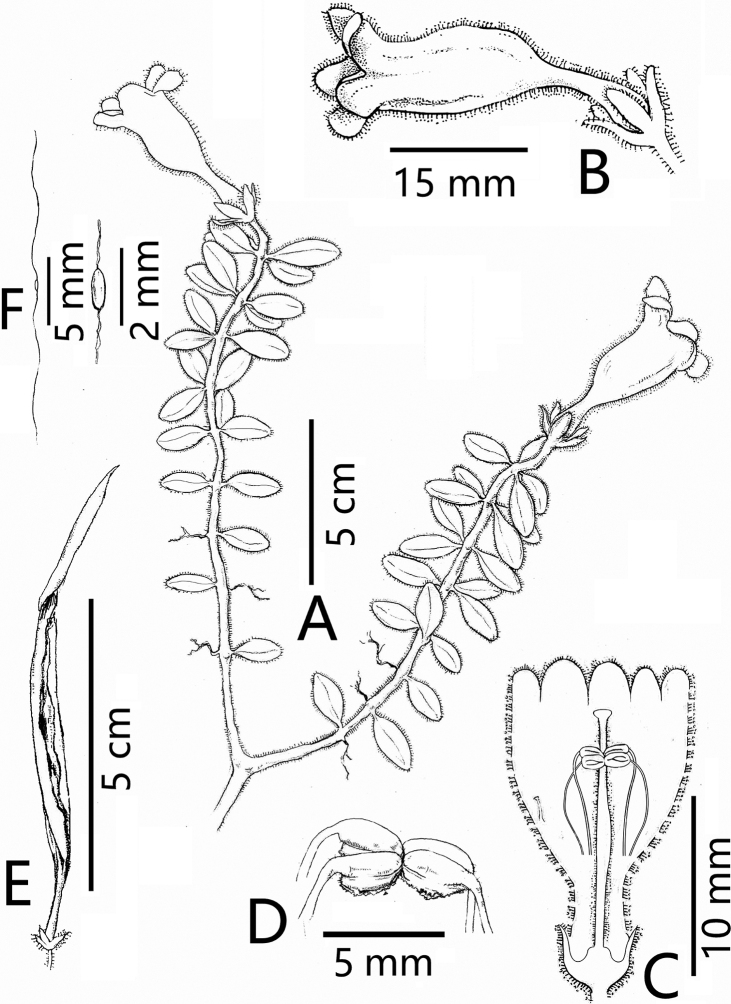
*Aeschynanthussmaragdinus* F.Wen & J.Q.Qin **A** habit **B** flower **C** flower dissection **D** fruit **E** seed **F** seed grain. **A–F** from Isotype. Scale bars: 1 cm (**A–E**); 1 mm (**F**). Illustrated by Rui-Feng Li.

**Figure 2.**
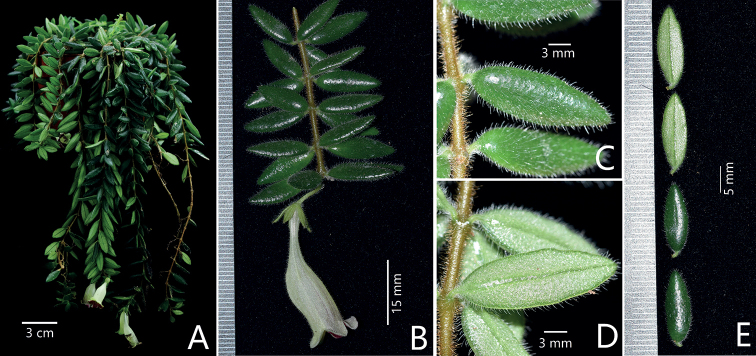
Plants *Aeschynanthussmaragdinus* F.Wen & J.Q.Qin **A** habit **B** a branch with a single flower **C** the adaxial surface of leaf blades and stem **D** the abaxial surface of leaf blades and stem **E** the adaxial and abaxial surfaces of leaf blades. Photographs by De-Chang Meng.

**Figure 3.**
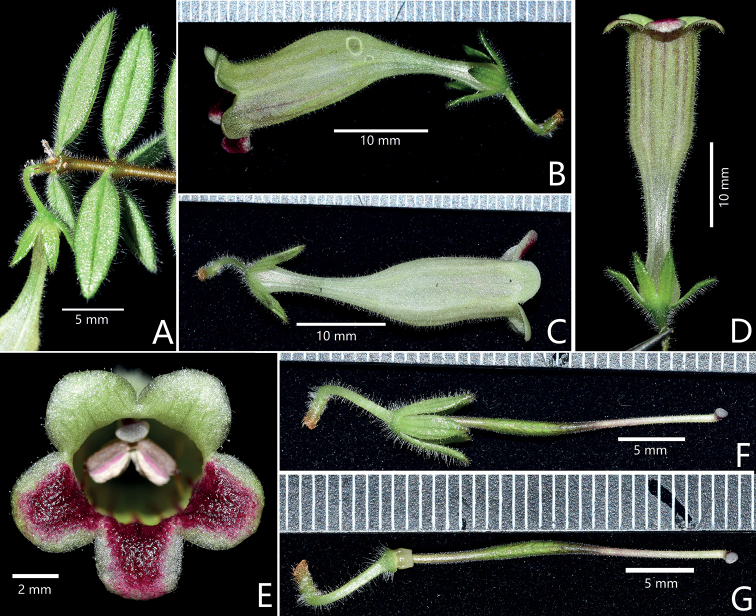
Propagative organs of *Aeschynanthussmaragdinus* F.Wen & J.Q.Qin (I) **A** a branch with terminal flower **B** lateral view of flower **C** top view of flower **D** upward view of flower **E** frontal view of corolla **F** pistil with calyx lobes **G** pistil without calyx lobes. Photographs by De-Chang Meng.

**Figure 4.**
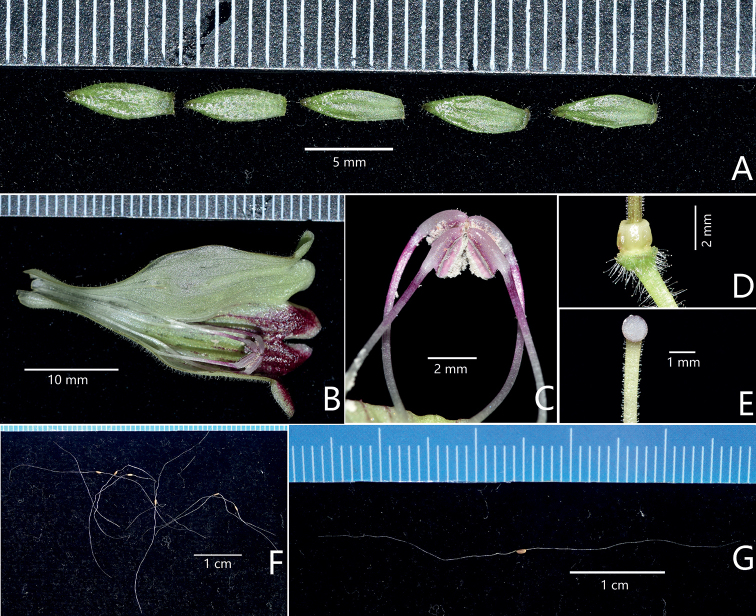
Propagative organs of *Aeschynanthussmaragdinus* F.Wen & J.Q.Qin (II) **A** calyx lobes (left two lobes showing abaxial surfaces; right three showing adaxial surfaces) **B** opened corolla **C** four fertile stamens **D** disk **E** stigma **F** seeds **G** seed. Photographs **A–E** by De-Chang Meng, **F, G** by Fang Wen.

**Figure 5.**
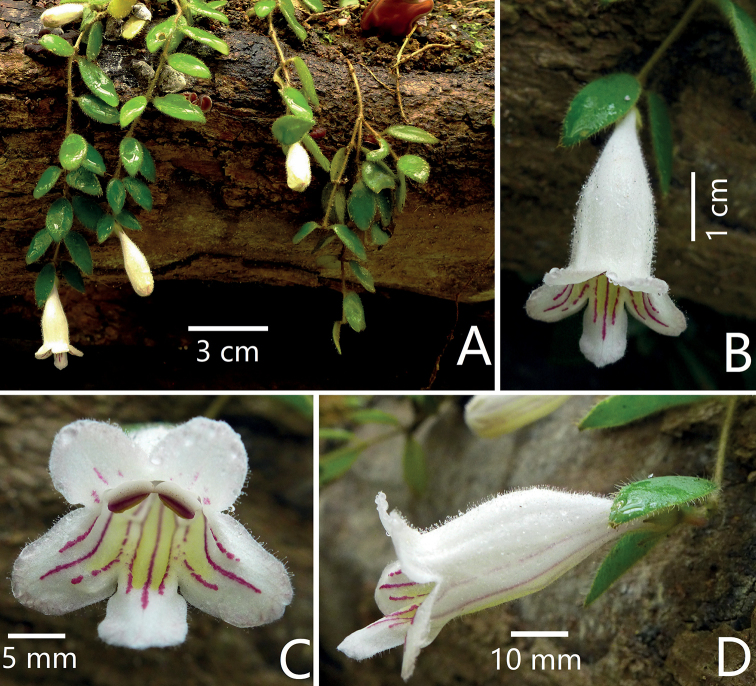
The morphologically similar species, *Aeschynanthuschiritoides* C.B.Clarke **A** habit **B** top view of flower **C** frontal view of corolla **D** lateral view of a flower. Photographs by Fang Wen.

**Table 1.** Detailed comparison of *Aeschynanthussmaragdinus* F.Wen & J.Q.Qin and its relative *A.chiritoides* C.B.Clarke.

Characters	*A.smaragdinus*	*A.chiritoides*
Phyllotaxis	in whorls of 3, sometimes opposite or in whorls of 4, occasionally three types on a single branch	opposite or in whorls of 3, often both types on a single branch
Pedicel indumentum	densely erectly glandular pubescent	densely villous
Calyx lobes indumentum	adaxially nearly glabrous, abaxially erectly glandular puberulent	adaxially and abaxially glandular- or eglandular-villous or a mixture of both
Corolla and limb lobes color	pale yellowish green to greenish; upper lobes pale green to yellowish green and lower lobes brownish red to maroon, with dark brownish red lines down into the tube	white or slightly yellowish or greenish with a few thin purple lines, all lobes white or slightly pale green with pale purplish lines
Disk	ca. 1.2 mm high and margin entire	1.8–3.6 mm high, 5-crenate or a simple annular ring
Seed grain size	ca. 1 × 0.5 mm	1.2–3 × ca. 0.3 mm
Seed appendages length	apical appendage, a filiform hair, ca. 25 mm long; hilar appendage, a single filiform hair, ca. 24 mm long	apical appendage a filiform hair, ca. 22 mm long; hilar appendage, a single filiform hair, ca. 17 mm long

#### Type.

China. Yunnan Province: Tengchong city, Mangbang township, 24°93'N, 98°67'E, altitude ca. 1500 m, April 20, 2017, *Jia-Qi Qin QJQ170420-01* (holotype IBK!; Isotype: KUN!)

#### Description.

Creeping or hanging subshrubs, epiphytic, with stem branched, greenish brown to pale brown, slender, with shorter internode (5–10 mm long), ca. 1 mm in diam., spreading rust-brown and white pubescent, occasionally roots at the node of the stem. Leaves in whorls of 3, sometimes opposite or in whorls of 4, occasionally three types on a single branch; petiole 0.5–1.0 mm long, green, sometimes subsessile, pubescent; leaf blade fleshy, thick, chartaceous when dried and size lessened, narrowly elliptic, elliptic to obovate and the cross-section olive-shaped, dark green on the adaxial surface and pale green on abaxial surface, not marbled, 1–1.9 × 0.6–1 cm, apex acute to subacute, base cuneate, adaxially and abaxially white pubescent; margin entire to slightly undulate; lateral veins invisible on both surfaces, main vein invisible on the adaxial surface but prominent and dark green on abaxial surface, secondary and tertiary venation obscure or invisible. Inflorescences usually terminal or subterminal, occasionally axillary, flowers solitary; peduncles almost obsolete, 0.5–1.0 mm long, ca. 1 mm in diam., slightly woody, arising from the axils, densely glandular-pubescent; bracts tiny and deciduous; pedicels 10–18 mm long, pubescent, pale green, densely erect glandular-pubescent. Calyx of 5 separate lobes free to base, campanulate, segments equal, both surfaces green, oblong-lanceolate to narrowly elliptic, ca. 6.0 × 1.5 mm, adaxially nearly glabrous, abaxially erect glandular puberulent, apex acute to subacute, margin entire. Corolla 30–35 mm long, externally green, pale yellowish green to greenish; upper lobes pale green to yellowish green, lower lobes pale green to yellowish green suffused pale brownish red, internally tube pale green, upper lobes pale green to yellowish green and lower lobes brownish red to maroon, with dark brownish red lines down into tube; corolla tube slightly obliquely swollen horn-shaped, often curved at the tube middle, ca. 2 cm long; ca. 7 mm in diam. at the mouth, the base of the corolla tube gibbose, and ca. 3 mm in diam.; limb distinctly 2-lipped, adaxial lip 2-lobed, lobes obliquely semicircular, spreading, ca. 4 mm in diam. at the base, apex rounded; abaxial lip 3-lobed from slightly below middle, 3 lobes bicolored, brownish red to maroon in the center and pale green on the edge of lobes, lateral lobes slightly obliquely oblong to oblong and the central one oblong, spreading, ca. 5 mm long, ca. 3.5 mm in diam. at the base, apex rounded; glandular puberulent outside, glabrous inside. Stamens 4, not exserted, all 4 fused together; filaments pale green from the middle to the base and gradually changing to pale purple from the middle to the top, glabrous, anthers pale purple; anterior filaments ca. 9 mm long, posterior filaments ca. 11 mm long, all adnate 14–15 mm above the corolla base; anthers ca. 2.5 × 1 mm, oblong, 2-locular, thecae parallel, dehiscing longitudinally, pollen pale yellow; Staminode 1, filiform, ca. 1 mm long, adnate to ca. 15 mm above the corolla base, glabrous. Disk annular, ca. 1.2 mm high, wax yellow, glabrous, margin entire. Pistil ca. 24 mm long; stipe 7–9 mm long; ovary narrowly spindly, ca. 1.5 cm long, glandular-puberulent, bicolor, brownish green at the base and top of the ovary but green with purplish stripes in the middle of the ovary; style ca. 9 mm long, extending out of corolla tube at the end of the single flowering phase, glandular-pubescent; stigma capitate, pale purple to whitish purple, ca. 1 mm in diam. Capsule linear, ca. 9 cm long, glabrous. Seed grain oblong-oval, ca. 1 × 0.5 mm, warty, apical appendage a filiform hair, ca. 25 mm long; hilar appendage a single filiform hair, ca. 24 mm long; appendages papillose.

#### Phenology.

Flowering in December to February, fruiting from April to June.

#### Etymology.

Compared with most other species of *Aeschynanthus*, the beautiful green leaves and flowers of this dwarf plant resemble an emerald. The specific epithet ‘*smaragdinus*’ is derived from the Latin vocabulary and means a unique dazzling green.

#### Vernacular name.

翡翠芒毛苣苔 (Chinese name); Féi Cuì Máng Máo Jù Tái (Chinese pronunciation).

#### Distribution and habitat.

Presently, *Aeschynanthussmaragdinus* is only found in the type locality, Mangbang Township, Tengchong City, Yunnan. The species grow on moist, shady tree trunk surfaces in a monsoon rainforest at ca. 1500 m. Thus, it enjoys a cool environment with high air humidity in a moderately shaded monsoon rainforest.

#### Conservation status.

*Aeschynanthussmaragdinus* is so far only known from the type locality. The total distribution area of this species is approximately five km^2^ with a population size of about 500 mature individuals. However, we consider the data incomplete, and the new species is categorized as ‘Data Deficient’ (DD) according to the IUCN criteria (IUCN 2022).

#### Notes.

The plant size of *Aeschynanthussmaragdinus* is dwarf, and the leaf blade length is less than 2 cm, but the flower length is from 3 cm to 3.5 cm, and the proportion of flowers and leaves is unusual in this genus. Besides this new taxon, other species have this property, for instance *A.chiritoides*, *A.gracilis* Parish & C.B.Clarke, *A.minutifolius* D.J.Middleton, *A.persimilis* Craib (Middleton 2007). However, the green flowers are especially distinctive. These characters differ from *A.chiritoides* in morphology (Table 1).

## Supplementary Material

XML Treatment for Aeschynanthus smaragdinus

## References

[B1] Cen HF Tan YH Wei YG Wen F (2017) New records of five species of angiosperms in Guangxi. Journal of Plant Resources and Environment 26(1): 119–120. 10.3969/j.issn.1674-7895.2017.01.19

[B2] Chen YS (2019) Flora of Pan-Himalaya: A Preliminary Catalogue of Vascular Plants in the Pan-Himalaya. Cambridge University Press & Science Press, Beijing, 371 pp.

[B3] Hu J Xiong YM Li L Liu Q Wen F (2020) Rediscovery of *Aeschynanthusmonetaria* (Gesneriaceae) in Southeast Tibet, China after more than 100 years. Phytotaxa 450(1): 109–114. 10.11646/phytotaxa.450.1.9

[B4] IUCN Standards and petitions committee (2022) Guidelines for Using the IUCN Red List Categories and Criteria. Version 15. Prepared by the Standards and petitions committee. https://www.iucnredlist.org/resources/redlistguidelines [accessed on 4 Mar. 2022]

[B5] Jack W (1823) [1825] On Cyrtandraceae, a new natural order of plants. Transactions of the Linnean Society of London 14(1): 23–45. 10.1111/j.1095-8339.1823.tb00078.x

[B6] Li ZY Wang YZ (2005) *Aeschynanthus* Jack. In: Li ZY Wang YZ (Eds) Plants of Gesneriaceae in China.Henan Science and Technology Publishing House, Zhenzhou, 363–381.

[B7] Mendum M Lassnig P Weber A Christie F (2001) Testa and seed appendage morphology in *Aeschynanthus* (Gesneriaceae): Phytogeographical patterns and taxonomic implications. Botanical Journal of the Linnean Society 135(3): 195–213. 10.1111/j.1095-8339.2001.tb01091.x

[B8] Middleton D (2007) A revision of *Aeschynanthus* (Gesneriaceae) in Thailand. Edinburgh Journal of Botany 64(3): 363–429. 10.1017/S0960428607004878

[B9] Middleton D (2009) A revision of *Aeschynanthus* (Gesneriaceae) in Cambodia, Laos and Vietnam. Edinburgh Journal of Botany 66(3): 391–446. 10.1017/S0960428609990047

[B10] Middleton D (2016) A revision of *Aeschynanthus* in Singapore and Peninsular Malaysia. Gardens’ Bulletin Singapore 68(1): 1–63. 10.3850/S2382581216000016

[B11] Olimpos SMB Mansibang JA (2021) *Aeschynanthusrejieae* (Gesneriaceae), a new species of lipstick vine from Tawi-Tawi, Philippines. Phytotaxa 487(1): 83–90. 10.11646/phytotaxa.487.1.7

[B12] Wang W Pan K Li ZY Weitzman AL Skog LE (1998) Gesneriaceae. In: Wu ZY Raven PH (Eds) Flora of China (Vol.18) (Scrophulariaceae through Gesneriaceae). Science Press, Beijing, and Missouri Botanical Garden Press, St. Louis, 375–385.

[B13] Weber A (2004) Gesneriaceae. In: Kubitzki K Kadereit JW (Eds) The Families and Genera of Vascular Plants (Vol.7). Flowering plants: Dicotyledons; Lamiales (except Acanthaceae including Avicenniaceae). Springer, Berlin & Heidelberg, 63–158. 10.1007/978-3-642-18617-2_8

[B14] Weber A Clark JL Möller M (2013) A new formal classification of Gesneriaceae. Selbyana 31(2): 68–94.

[B15] Wei YG (2018) The Distribution and Conservation Status of Native Plants in Guangxi, China. China Forestry Publishing House, Beijing, 908 pp.

[B16] Wei YG Do VT Wen F (2022) A Checklist to the Plants of Northern Vietnam. China Forestry Publishing House, Beijing, 606 pp.

